# *Aedes aegypti* abundance and insecticide resistance profiles in the Applying *Wolbachia* to Eliminate Dengue trial

**DOI:** 10.1371/journal.pntd.0010284

**Published:** 2022-04-20

**Authors:** Warsito Tantowijoyo, Stephanie K. Tanamas, Indah Nurhayati, Sigit Setyawan, Nida Budiwati, Iva Fitriana, Inggrid Ernesia, Dwi Satria Wardana, Endah Supriyati, Eggi Arguni, Yeti Meitika, Equatori Prabowo, Bekti Andari, Benjamin R. Green, Lauren Hodgson, Edwige Rancès, Peter A. Ryan, Scott L. O’Neill, Katherine L. Anders, M. Ridwan Ansari, Citra Indriani, Riris Andono Ahmad, Adi Utarini, Cameron P. Simmons

**Affiliations:** 1 World Mosquito Program Yogyakarta, Centre for Tropical Medicine, Faculty of Medicine, Public Health and Nursing, Universitas Gadjah Mada, Yogyakarta, Indonesia; 2 World Mosquito Program, Institute of Vector-borne Disease, Monash University, Clayton, Australia; 3 Department of Child Health, Faculty of Medicine, Public Health and Nursing, Universitas Gadjah Mada, Yogyakarta, Indonesia; 4 Department of Biostatistics, Epidemiology and Public Health, Faculty of Medicine, Public Health and Nursing, Universitas Gadjah Mada, Yogyakarta, Indonesia; 5 Department of Health Policy and Management, Faculty of Medicine, Public Health and Nursing, Universitas Gadjah Mada, Yogyakarta, Indonesia; 6 Oxford University Clinical Research Unit, Hospital for Tropical Diseases, Ho Chi Minh City, Vietnam; University of Florida, UNITED STATES

## Abstract

The Applying *Wolbachia* to Eliminate Dengue (AWED) trial was a parallel cluster randomised trial that demonstrated *Wolbachia* (*w*Mel) introgression into *Ae*. *aegypti* populations reduced dengue incidence. In this predefined substudy, we compared between treatment arms, the relative abundance of *Ae*. *aegypti* and *Ae*. *albopictus* before, during and after *w*Mel-introgression. Between March 2015 and March 2020, 60,084 BG trap collections yielded 478,254 *Ae*. *aegypti* and 17,623 *Ae*. *albopictus*. Between treatment arms there was no measurable difference in *Ae*. *aegypti* relative abundance before or after *w*Mel-deployments, with a count ratio of 0.96 (95% CI 0.76, 1.21) and 1.00 (95% CI 0.85, 1.17) respectively. More *Ae*. *aegypti* were caught per trap per week in the *w*Mel-intervention arm compared to the control arm during *w*Mel deployments (count ratio 1.23 (95% CI 1.03, 1.46)). Between treatment arms there was no measurable difference in the *Ae*. *albopictus* population size before, during or after *w*Mel-deployment (overall count ratio 1.10 (95% CI 0.89, 1.35)). We also compared insecticide resistance phenotypes of *Ae*. *aegypti* in the first and second years after *w*Mel-deployments. *Ae*. *aegypti* field populations from *w*Mel-treated and untreated arms were similarly resistant to malathion (0.8%), permethrin (1.25%) and cyfluthrin (0.15%) in year 1 and year 2 of the trial. In summary, we found no between-arm differences in the relative abundance of *Ae*. *aegypti* or *Ae*. *albopictus* prior to or after *w*Mel introgression, and no between-arm difference in *Ae*. *aegypti* insecticide resistance phenotypes. These data suggest neither *Aedes* abundance, nor insecticide resistance, confounded the epidemiological outcomes of the AWED trial.

## Author summary

Dengue is a mosquito-borne viral disease and a major public health problem in the tropical and subtropical world. It is caused by any of the four dengue virus serotypes. In a previously published randomised clinical trial, called the AWED trial, we demonstrated that releases of *Aedes aegypti* mosquitoes infected with the insect bacterium *Wolbachia* can reduce the case incidence of dengue by 77%. In this current study, we compared the abundance of *Ae*. *aegypti* mosquitoes in the neighbourhoods where *Wolbachia*-infected mosquitoes were released versus the untreated neighbourhoods. This was important to do so that scientists could understand the mechanism for how *Wolbachia* releases reduced dengue incidence. Between March 2015 and March 2020, we did not observe any differences in *Ae*. *aegypti* abundance before or after *Wolbachia*-deployments in the AWED trial area. There was also no difference in the abundance of the related mosquito, *Ae*. *albopictus*, before, during or after *w*Mel-deployment. We also compared insecticide resistance characteristics amongst *Ae*. *aegypti* in the first and second years after *Wolbachia* -deployments and found no difference between mosquitoes from *Wolbachia*-treated and untreated neighbourhoods. These data suggest neither *Aedes* abundance, nor insecticide resistance, were confounding sources to the epidemiological outcomes of the AWED trial.

## Introduction

Dengue is the most common mosquito-borne viral infection worldwide and is caused by any of the four serotypes of dengue virus (DENV). *Aedes aegypti* mosquitoes are the primary vectors of dengue viruses between humans. An estimated 4 billion people in 128 countries are at risk from local DENV transmission and the geographic range and disease burden have expanded substantially in the past 50 years [1].

*Wolbachia* bacteria are endosymbionts of insects that are present in several mosquito species including *Aedes albopictus* and *Culex pipiens*, but not in *Ae*. *aegypti*. *Ae*. *aegypti* stably transinfected with the *Wolbachia* strain *w*Mel possess three traits that make them attractive as a public health intervention against dengue and other medically important arboviruses. First, *w*Mel infection renders *Ae*. *aegypti* resistant to disseminated infection by dengue, Zika and chikungunya viruses [2–4]. Second, female mosquitoes vertically transmit *w*Mel with high fidelity to their offspring, thus ensuring population-level *Wolbachia* establishment persists for many years [5–7]. Third, *w*Mel manipulates mosquito reproductive outcomes, a process called cytoplasmic incompatibility (CI), to favour its own population introgression [5]. Combined, these traits enable the application of *w*Mel as a public health intervention to reduce the vectorial capacity of mosquito populations to transmit DENV between humans.

The Applying *Wolbachia* to Eliminate Dengue (AWED) cluster randomised trial introgressed *w*Mel into the *Ae*. *aegypti* population in 12 geographic clusters in Yogyakarta, Indonesia. Over 27 months, the incidence of virologically-confirmed dengue was reduced by 77% and the incidence of dengue hospitalisations by 86% when compared to untreated (control) clusters [8,9]. The most parsimonious explanation for the reduction in dengue incidence in the *w*Mel introgressed clusters is the reduced vector competence of *w*Mel-infected mosquito population. However, it’s plausible that *w*Mel introgression had other effects on the *Ae*. *aegypti* population in Yogyakarta that contributed to these epidemiological outcomes. For example, *w*Mel-infection decreases the survivorship of mosquito eggs [10–13] and this could plausibly result in a smaller *Ae*. *aegypti* population size in geographic clusters where *w*Mel was introgressed. Periods of self-incompatibility represent another route to a smaller *Ae*. *aegypti* population size—this could occur if maternal transmission of *w*Mel was periodically imperfect or CI transiently broke down [14,15]. Additionally, insecticide resistance characteristics in the *w*Mel-infected mosquito population might change over time compared to wild-type mosquitoes in control clusters because of differences in selective pressure from insecticide use in the community.

Here we report longitudinal comparisons of *Ae*. *aegypti* relative abundance and insecticide resistance characteristics between *w*Mel-intervention clusters and control clusters. We also report on the relative abundance and distribution of the secondary vector *Ae*. *albopictus* between intervention clusters and control clusters. These comparisons were prespecified secondary endpoints in the AWED protocol [9].

## Methods

### Study design

The AWED study was a parallel two-arm non-blinded cluster randomised controlled trial conducted in a single site in Yogyakarta City, Indonesia. The 26km^2^ study site with a population of 312,000 people was subdivided into twenty-four contiguous clusters approximately 1km^2^ in size (range 0.7km^2^-1.65km^2^). Twelve clusters were randomly selected to receive *Wolbachia* deployments and 12 were untreated. There are no buffer areas between clusters, but natural borders (roads, rivers, non-residential areas) were used to define cluster boundaries as much as possible, to limit the spatial spread of *w*Mel from treated clusters into untreated areas, and of wild-type mosquitoes into *w*Mel-treated clusters. No attempt was made to alter the routine dengue prevention and vector control activities conducted by public and private agencies throughout the study area (treated and untreated clusters).

### *Wolbachia* deployment

*w*Mel-infected *Ae*. *aegypti* were released as eggs using mosquito release containers (MRCs), as described previously [8]. Releases occurred between March and December 2017, with 9–14 rounds of releases in each intervention cluster. Releases stopped in each cluster when the prevalence of *Wolbachia* in field-caught mosquitoes was >60% for three consecutive release weeks. MRCs were reset every two weeks. An MRC was placed in 1–2 randomly selected locations within each 50x50m grid square across the intervention area. Permission was obtained from property owners to place MRCs on private property.

### Mosquito surveillance

Weekly surveillance of *Ae*. *aegypti* in Yogyakarta commenced in March 2015, two years prior to the AWED trial releases, and continued until the trial’s conclusion in March 2020. Mosquitoes were collected using a network of BG Sentinel traps (Biogents, Germany) set indoors in residences throughout the city. Each trap was equipped with an accumulator to sustain power in case of a power outage. Written consent was obtained from heads of households hosting BG traps. The BG trap network had three temporal windows; the pre-release period, the release period during which *w*Mel mosquitoes were released and the post-release period. For reasons related to budget and workforce capacity, it was not possible to keep the BG trap density constant between each phase but we did ensure balance between treatment arms for the great majority of the 5-year observation period. The median trapping density in the pre-release period March 2015—February 2017 was 3.5 traps/km^2^ (range 0.9 to 23.3) in the intervention clusters and 5.2 traps/km^2^ (range 0.6 to 6.8) in the untreated clusters. During the *w*Mel release period March—December 2017, the median trapping density in the intervention clusters was increased to 15.7 traps/km^2^ (range 10.2 to 18.2). For the first seven weeks of releases the trapping density in the untreated clusters remained the same (median trapping density 5.2 traps/km^2^ [range 0.9 to 6.3]), followed by no trapping for two weeks. From May 2017 the number of traps in the untreated clusters was increased for the remainder of the release period to a median of 14.8 traps/km^2^ (range 5.9 to 16.8). In the post-intervention period January 2018—March 2020, a fixed spatial distribution of BG traps, with comparable density between study arms, was maintained. The median was 15.9 traps/km^2^ (range 12.3 to 18.1) in the intervention clusters and 14.8 traps/km^2^ (range 8.0 to 16.8) in the untreated clusters; the median (range) distance of traps from the cluster boundary was 68 (42–102) metres in the intervention clusters and 68 (32–84) metres in untreated clusters; and the median (range) of the average distance to the nearest trap was 211 (185–239) metres in intervention clusters and 225 (203–280) metres in untreated clusters. The median (range) nearest neighbour index was 1.6 (1.4–1.9) in the intervention clusters and 1.7 (1.5–1.9) in the untreated clusters, where a value of <1 indicates a clustered pattern and >1 indicates a dispersed pattern. The post-intervention BG trap distribution per cluster is shown in S1 Fig and tabulated in S1 Table.

Mosquitoes captured in BG traps were demobilised at -20°C for ≥1 hour, then identified by morphological features. The number of mosquitoes caught in each BG trap was recorded by species and sex. *Ae*. *aegypti* were stored at -20°C in 80% ethanol until testing for *w*Mel infection. Individual whole mosquitoes were homogenised in 96 well plates using 1mm glass beads and a bead beater. After centrifugation, an aliquot of the homogenate supernatant was used for detection of *w*Mel *Wolbachia* by qualitative PCR Taqman assay on a Roche LightCycler 480. The qPCR conditions consisted of a denaturation step at 95°C for 5 minutes followed by 45 cycles of PCR (denaturation at 95°C for 10 seconds, annealing at 60°C for 15 seconds, and extension at 72°C for 1 second with the single acquisition) followed by a cooling down step at 40°C for 10 seconds. Specific primers targeting the gene encoding *Ae*. *aegypti Rps17* and *w*Mel *WD0513* were used as previously described [16], but with replacement of the Cy5-BHQ3 fluorophore-quencher pair in the *w*Mel probe with the fluorophore-quencher LC640-IowaBlack (Integrated DNA technologies) [17]. Testing was at weekly intervals when *Wolbachia* prevalence was <80% and 4-week intervals when establishment was achieved (≥80% cluster level prevalence for two consecutive testing weeks).

### Insecticide resistance testing

Insecticide resistance testing by bioassay was performed in 2018 and 2019 on field-derived adults sourced from each of the 24 clusters. Each cluster generated a cohort of mosquitoes that was tested for insecticide resistance, thus there were 12 cohorts from *w*Mel-treated clusters and 12 from untreated clusters each year, with the exception of 2018 when there were 11 cohorts from *w*Mel-treated clusters. Cyfluthrin and malathion were chosen for testing on the basis of their use in fine aerosol (fogging) spraying by the Yogyakarta Department of Health in response to dengue cases in the community. Permethrin was included on the basis of it being representative of pyrethroids commonly found in household insecticide sprays. Mosquito eggs were collected using ovitraps from in and around residential properties that hosted BG traps for *Wolbachia* monitoring. To maximise the probability that ovitraps were attracting only the desired populations of mosquitoes (i.e. 100% *w*Mel infected or 100% wild-type) they were placed in properties where recent BG trap surveillance indicated 0% *w*Mel prevalence (in untreated clusters) or 100% *w*Mel prevalence (in intervention clusters). In 2018, a total of 346 ovitraps were deployed in BG host houses for egg collection over three weeks. In 2019, a total of 345 ovitraps were deployed in BG host houses and 370 in a neighbouring house for egg collection over two weeks. Neighbouring houses were added in 2019 to boost egg collection as there was some difficulty in getting sufficient eggs from only BG host houses in 2018. In 2018 egg collections were performed in June-July and in 2019 collections were performed in September. Eggs were dried for 1 day, stored, and hatched when required. Eggs from the same cluster were pooled before hatching. *Ae*. *aegypti* mosquitoes were visually identified and selected during the larval stage. Female *Ae*. *aegypti* mosquitoes were selected at the pupal stage. In 2018, F0 mosquitoes (i.e. the adults derived from field collected eggs) were used directly for insecticide resistance testing. In 2019, F1 adults (i.e. the offspring of F0 adults) were used for insecticide resistance testing. The move to using F1 adults was to reduce the resource-intensive collection of sufficient eggs from the field. Insecticide type and concentrations used were in line with recommendations for *Ae*. *aegypti* mosquitoes and followed the WHO standard bioassay method, noting that this method is only semi-quantitative [18]. Insecticide impregnated papers were purchased from the WHO Collaborating Centre at the Universiti Sains Malaysia, Penang, Malaysia. The papers used were Malathion 0.8%, Permethrin 1.25%, and Cyfluthrin 0.15%. Pyrethroid control paper was used for Permethrin and Cyfluthrin, and OP-Carbamate control was used for Malathion. In each year, six rounds of insecticide resistance testing were conducted, with two intervention clusters and two untreated clusters randomly allocated to each round. The first three testing rounds in 2018 (covering 12 clusters) included four replication tubes for each insecticide and two negative control tubes, with 25 female *Ae*. *aegypti* mosquitoes per tube. The mosquitoes were three to five days old, fed with sugar only. Mosquitoes were kept in a paper-free tube for one hour to adapt, transferred to the tube containing insecticide-impregnated paper for one hour, then transferred back to the holding tube, with access to sugar solution, for 24 hours. Dead and live mosquitoes were counted after 24 hours. The Rockefeller *Ae*. *aegypti* strain was used as a susceptible control line in the 2019 experiments.

### Statistical analysis

The number of mosquitoes caught over time was summarised as the mean count per BG trap per week, by species and study arm (S1 Data). The ratio of female to male *Ae*. *aegypti* and *Ae*. *albopictus* over time was calculated on the basis of summed weekly counts and stratified by study arm. The ratio of *Ae*. *aegypti* to *Ae*. *albopictus* mosquitoes over time was also calculated on the basis of summed weekly counts and stratified by study arm.

Mixed-effects negative binomial regression was used to compare the number of *Ae*. *aegypti* caught per trap per week in the intervention arm versus the untreated arm, with the inclusion of BG trap as a random effect to account for clustering at the trap level. Zero-inflated negative binomial regression was used to compare the number of *Ae*. *albopictus* caught per trap per week in the intervention arm compared to the untreated arm, with a clustered sandwich estimator to control for clustering at the trap level. A zero-inflated model was used as >80% of trap collections had zero *Ae*. *albopictus*. All analyses included a binary indicator for dry/wet season as a covariate (wet season = November—April). The count ratio produced by the negative binomial regression model is the ratio of the mean number of *Ae*. *aegypti* or *Ae*. *albopictus* mosquitoes caught per trap per week in the intervention clusters compared to the untreated clusters. Data was analysed for the total observation period as well as separated by release status: pre-*w*Mel release, during release, and post-*w*Mel release, as defined earlier. Analysis of the total observation period was done with the inclusion of an indicator for release status as a covariate. The first nine weeks of releases were excluded from all analyses due to unequal trapping density by study arm and concerns that lower trapping density in the untreated clusters could result in less precise estimates of mosquito abundance compared to the intervention clusters.

Insecticide resistance was expressed as percentage mortality of *Ae*. *aegypti* exposed to various insecticides, calculated as the number of dead mosquitoes divided by the total number of mosquitoes in each replicate tube. The cluster-level percentage mortality is then the mean percentage mortality of the 4 replicate tubes in each cluster. Wilcoxon rank-sum test was used to compare the distribution of percentage mortality among 12 intervention clusters and 12 untreated clusters. Analysis was done separately for 2018 and 2019. One cluster from the intervention arm was excluded from analysis in 2018 due to low mosquito hatching rates resulting in insufficient mosquitoes for analysis.

All analyses were done using Stata version 16.0 (StataCorp, College Station, TX).

### Sensitivity analysis

To account for *Wolbachia* contamination into untreated clusters, a sensitivity analysis was performed comparing the number of *Ae*. *aegypti* and *Ae*. *albopictus* caught per trap per week in the intervention arm versus the untreated arm, with untreated clusters reclassified as ‘treated’ when the cluster level *Wolbachia* frequency is >50% for 2 monthly monitoring events within a 6-month rolling window and >50% of the BG traps in the cluster have detected *Wolbachia* during those monitoring events. Two untreated clusters were reclassified as intervention clusters due to *Wolbachia* contamination, one from September 2019 onwards and the other from February 2020 onwards.

### Power calculations

Power to detect a difference in mosquito abundance between *w*Mel intervention and untreated arms was calculated using a simulation with 1,000 repeats of *Ae*. *aegypti* monitoring data from the pre-*w*Mel release period. For each simulation, 50% of all BG traps were randomly assigned to the intervention arm and 50% to the untreated arm. The number of *Ae*. *aegypti* caught in traps assigned to the intervention arm was reduced by 10–40% in 2% increments, after first inflating by a factor of 1,000 to avoid non-integer values. A mixed-effect negative binomial regression was used to calculate the test statistic and p-value. The estimated power to detect a given effect size was determined as the proportion of observations (out of 1,000) for which the p-value was <0.05. The simulation indicated that we have 80% power to detect a difference in the number of *Ae*. *aegypti* of ≥20% between study arms, and 90% power to detect a difference of ≥23%.

## Results

### *w*Mel introgression in treated clusters

Between March 2017 and December 2017, *w*Mel deployments were performed in 12 of the 24 clusters of the AWED trial area as previously described [8]. *w*Mel introgressed rapidly and stably in each intervention cluster (S2A Fig). *w*Mel introgression (effectively contamination) was evident in some of the untreated clusters in 2019 and 2020 (S2B Fig).

### *Aedes aegypti* relative abundance before and after *w*Mel-deployments

**Fig 1:**
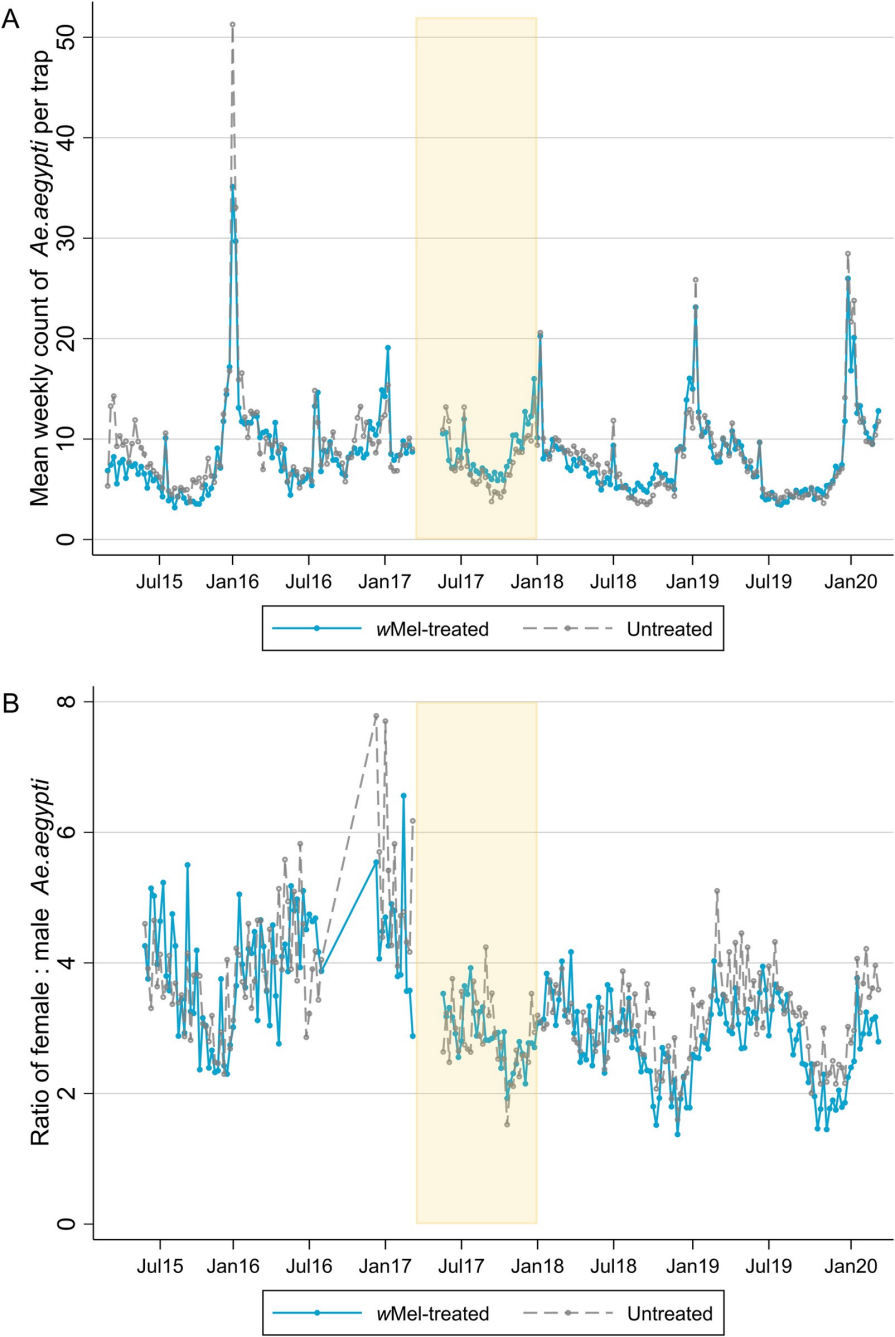
*Ae*. *aegypti* mosquitoes caught per week by study arm. Panel A is the mean number of *Ae*. *aegypti* mosquitoes caught per BG trap per week and panel B is the ratio of female to male *Ae*. *aegypti* caught. Releases of *w*Mel-infected *Ae*. *aegypti* mosquitoes occurred from March 2017 –December 2017 (yellow shading). The first nine weeks of releases are excluded from analyses due to unequal trapping density in the treated and untreated clusters. There was an average of 4 BG traps/km^2^ in *w*Mel-treated clusters and 5 BG traps/km^2^ in untreated clusters in the pre-release period, and 16 BG traps/km^2^ and 15 BG traps/km^2^ during and post-release. The warm, wet season in Yogyakarta is from November to April.

**Fig 2:**
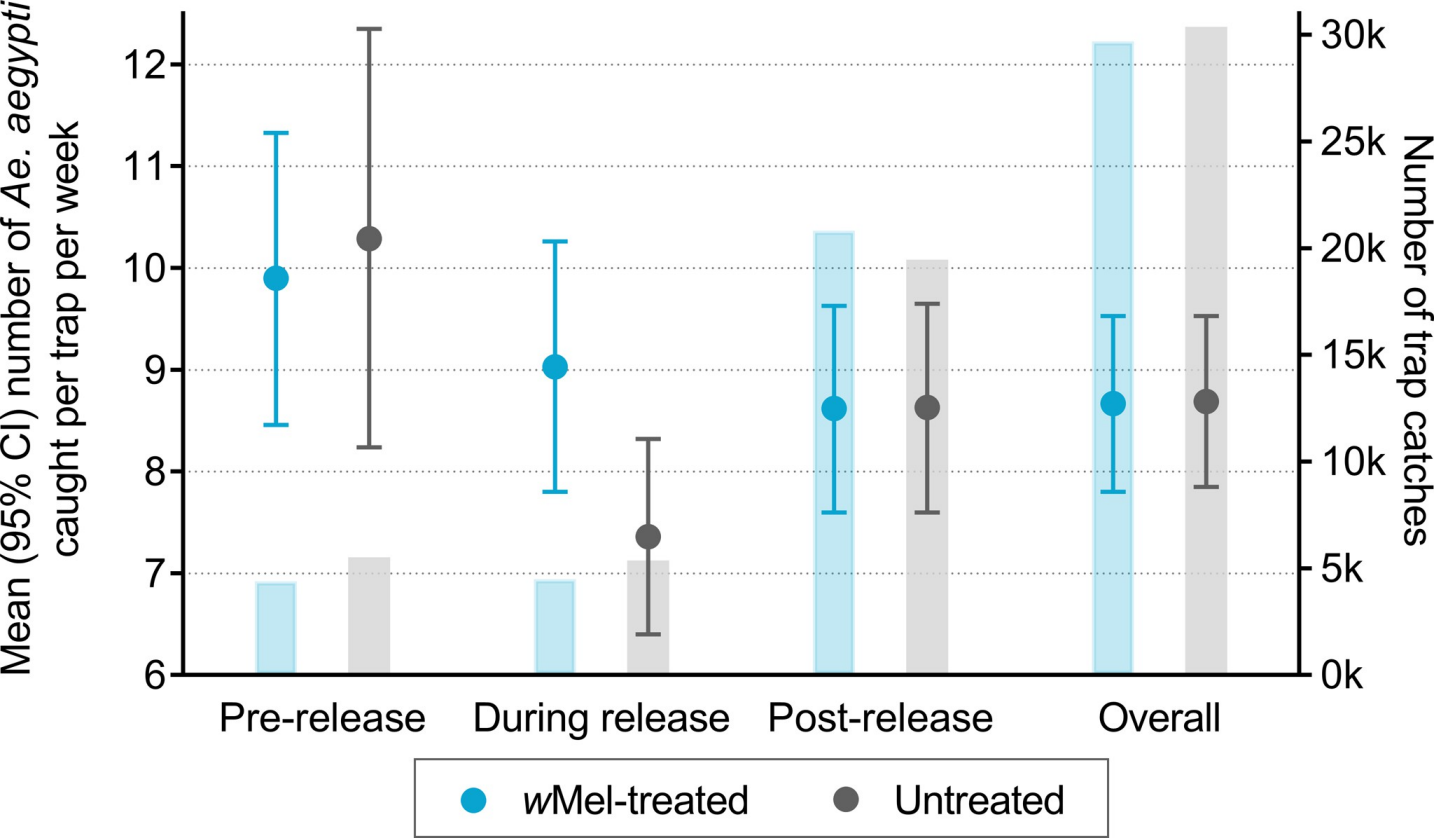
Mean number of *Ae*. *aegypti* mosquitoes caught per trap per week by study arm (circles) adjusted for season (wet/dry). Analysis of the total study period was additionally adjusted for release status (pre-release [March 2015 –February 2017], during release [March 2017 –December 2017], post-release [January 2018 –March 2020]). Bars are the number of trap collections in each arm. The first nine weeks of releases were excluded from analyses due to unequal trapping density in the treated and untreated clusters. There was an average of 4 BG traps/km^2^ in *w*Mel-treated clusters and 5 BG traps/km^2^ in untreated clusters in the pre-release period, and 16 BG traps/km^2^ and 15 BG traps/km^2^ during and post-release. More *Ae*. *aegypti* were caught per trap per week in the *w*Mel-treated arm compared to the untreated arm during *w*Mel deployments (count ratio 1.23 (95% CI 1.03, 1.46)). When formally compared, *Ae*. *aegypti* abundance did not differ between the *w*Mel-treated and untreated arm before (count ratio 0.96 (95% CI 0.76, 1.21), p = 0.74) or after *w*Mel-deployments (count ratio 1.00 (95% CI 0.85, 1.17), p = 0.99). Similar results were found for the post-intervention period when *w*Mel contamination was accounted for in a sensitivity analysis (count ratio 0.94 (95% CI 0.86, 1.03), p = 0.18).

A total of 60,084 BG trap collections yielded 478,254 *Ae*. *aegypti* and 17,623 *Ae*. *albopictus* mosquitoes in the area of the AWED cluster randomised trial in Yogyakarta City between March 2015 and March 2020. Fig 1A illustrates that the mean number of *Ae*. *aegypti* caught per trap each week in *w*Mel-treated versus untreated clusters was similar throughout this time period. The temporal trends in mean *Ae*. *aegypti* counts, peaking in January, are most likely due to climate factors, i.e. the warm rainy season producing abundant mosquito breeding sites in Yogyakarta between November to April. The ratio of female to male *Ae*. *aegypti* caught per week was also similar between *w*Mel-treated and untreated arms during the observation period (Fig 1B). Unsurprisingly, slightly more *Ae*. *aegypti* were caught per trap per week in the *w*Mel-treated arm compared to the untreated arm during deployments of *w*Mel-infected mosquitoes (count ratio 1.23 (95% CI 1.03, 1.46)) (Fig 2).

### *Aedes albopictus* relative abundance before, during and after *w*Mel-deployments

**Fig 3:**
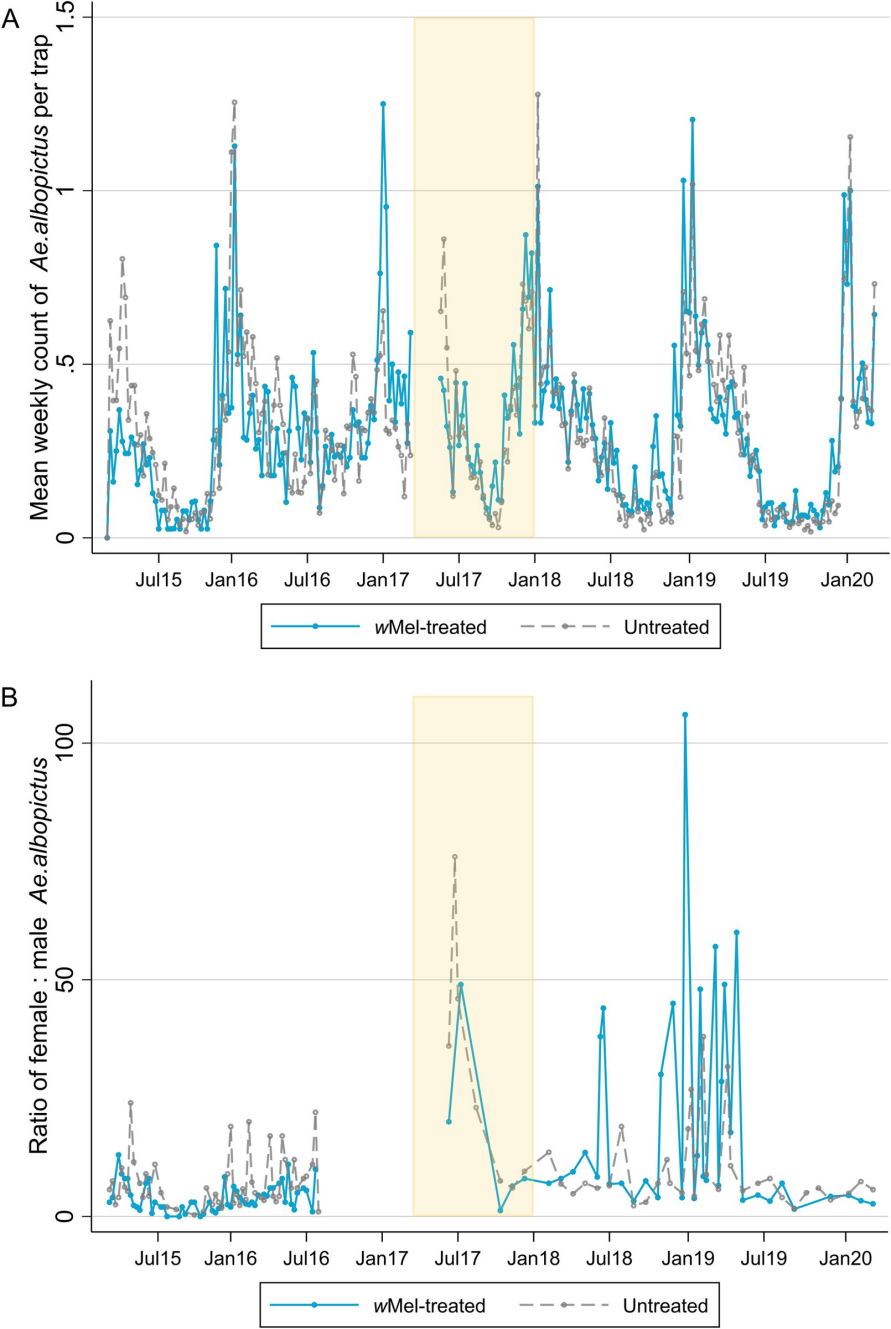
*Ae*. *albopictus* mosquitoes caught per week by study arm. Panel A is the mean number of *Ae*. *albopictus* mosquitoes caught per BG trap per week and panel B is the ratio of female to male *Ae*. *albopictus* caught. Releases of *w*Mel-infected *Ae*. *aegypti* mosquitoes occurred from March 2017 –December 2017 (yellow shading). The first nine weeks of releases are excluded from analyses due to unequal trapping density in the treated and untreated clusters. No male and very few female *Ae*. *albopictus* mosquitoes (median (interquartile range) 0 (0–0), range 0–97) were caught between mid-2016 and mid-2017. There was an average of 4 BG traps/km^2^ in *w*Mel-treated clusters and 5 BG traps/km^2^ in untreated clusters in the pre-release period, and 16 BG traps/km^2^ and 15 BG traps/km^2^ during and post-release.

**Fig 4:**
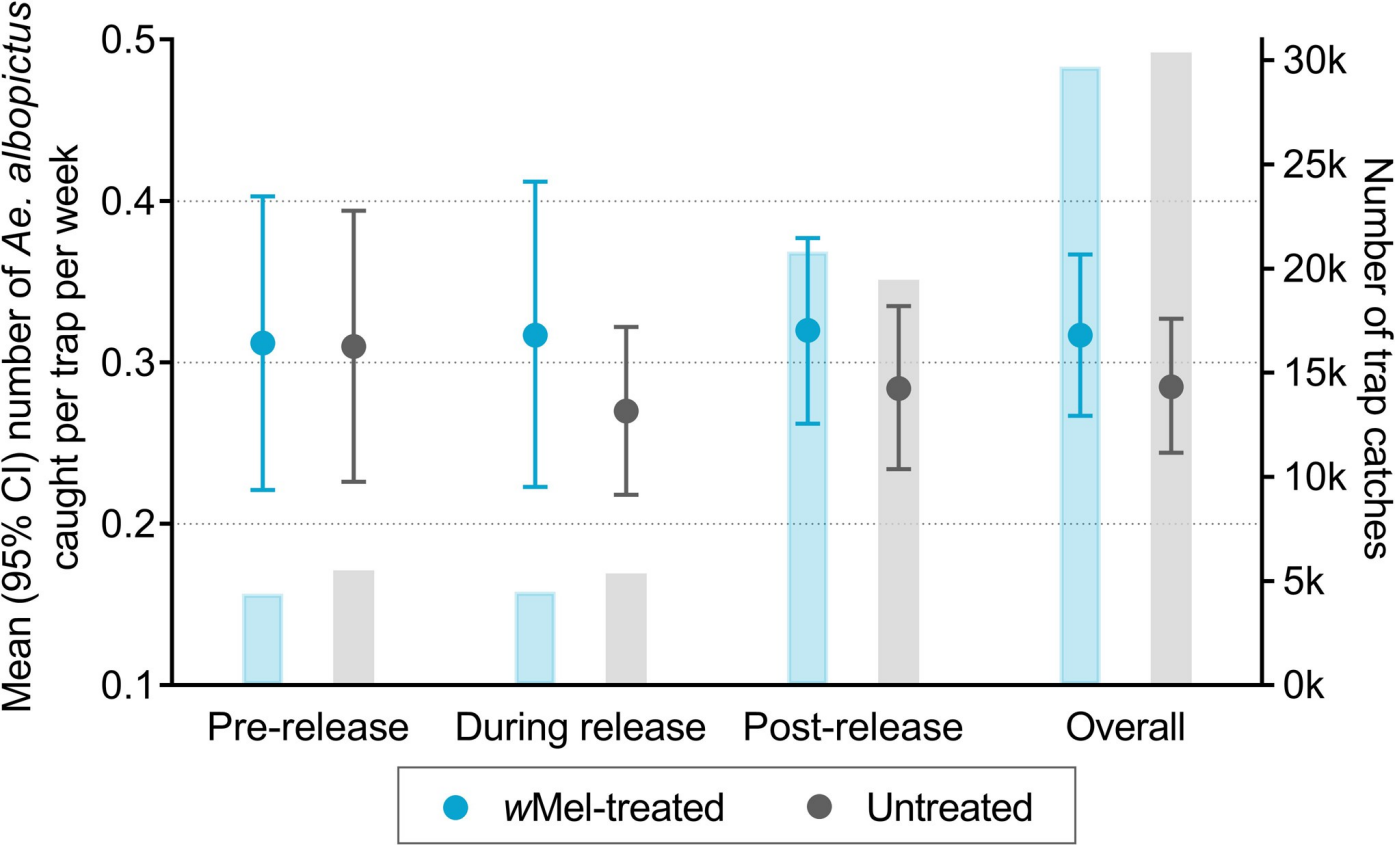
Mean number of *Ae*. *albopictus* mosquitoes caught per trap per week by study arm (circles) adjusted for season (wet/dry). Analysis of the total study period was additionally adjusted for release status (pre-release [March 2015 –February 2017], during release [March 2017 –December 2017], post-release [January 2018 –March 2020]). Bars are the number of trap catches in each arm. The first nine weeks of releases were excluded from analyses due to unequal trapping density in the treated and untreated clusters. There was an average of 4 BG traps/km^2^ in *w*Mel-treated clusters and 5 BG traps/km^2^ in untreated clusters in the pre-release period, and 16 BG traps/km^2^ and 15 BG traps/km^2^ during and post-release. There was no significant difference in *Ae*. *albopictus* abundance before (count ratio 1.00 (95% CI 0.68, 1.49)), during (1.08 (0.78, 1.51)) or after (1.12 (0.87, 1.45)) *w*Mel-deployments.

Fig 3A illustrates that the mean number of *Ae*. *albopictus* caught per trap each week in *w*Mel treated versus untreated clusters was similar between March 2015 and March 2020. The ratio of female to male *Ae*. *albopictus* caught per week was also similar between treatment arms (Fig 3B). There were no male *Ae*. *albopictus* mosquitoes caught between mid-2016 and mid-2017 and very few female *Ae*. *albopictus* (median (interquartile range) 0 (0–0), range 0–97). When formally compared, *Ae*. *albopictus* abundance did not differ by study arm before, during or after *w*Mel-deployment (Fig 4), with an overall average of 0.32 *Ae*. *albopictus* caught per trap per week (95% CI 0.27, 0.36) in the intervention arm and 0.29 (95% CI 0.25, 0.33) in the untreated arm (count ratio 1.11 (95% CI 0.90, 1.38), p = 0.33). Similar results were found when *Wolbachia* contamination in *Ae*. *aegypti* was accounted for, i.e. untreated clusters were reclassified as ‘treated’ as per the criteria described in the Methods section (count ratio 1.10 (95% CI 0.89, 1.36), p = 0.38).

### *Aedes aegypti* abundance relative to *Aedes albopictus*

**Fig 5:**
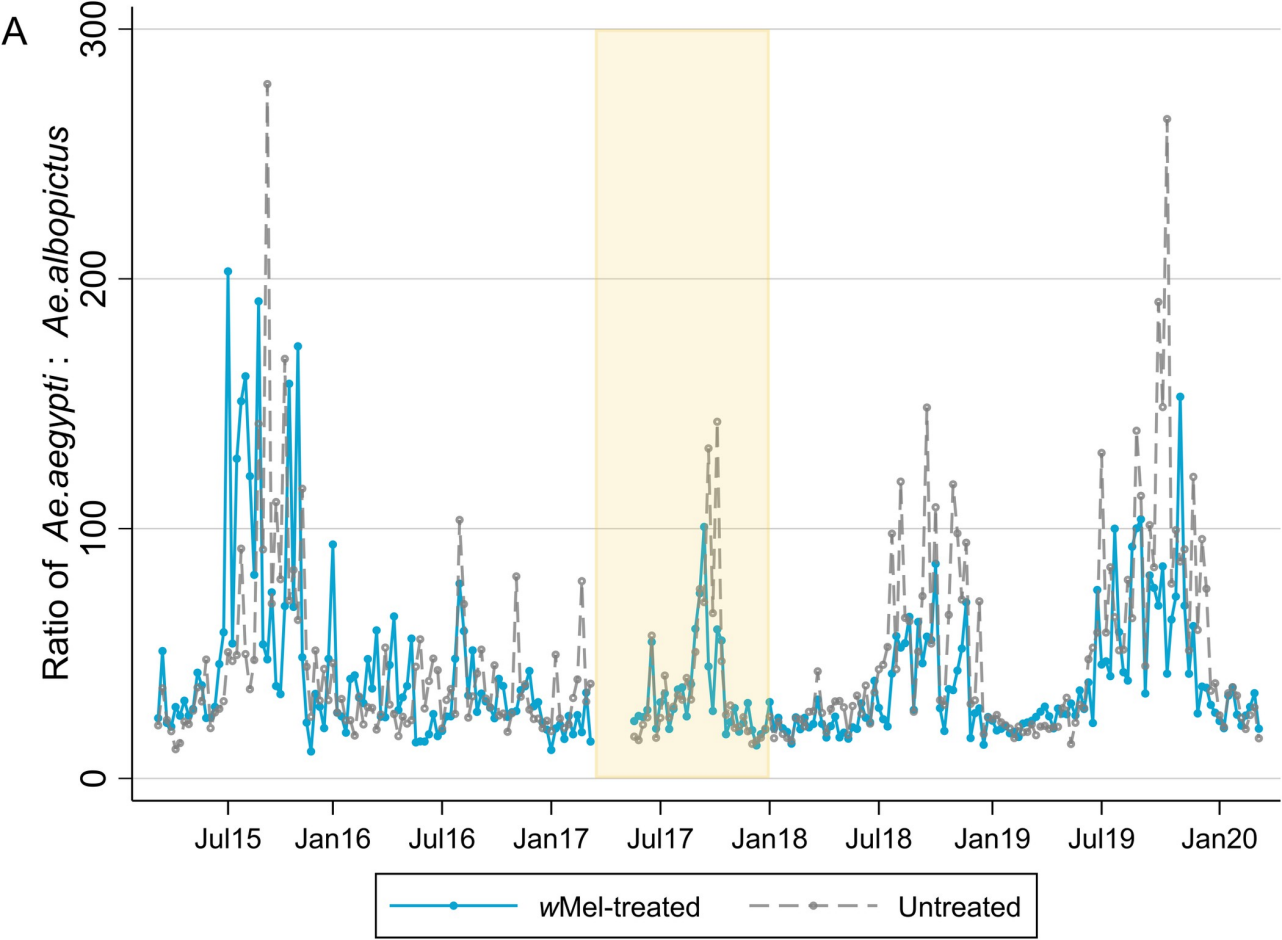
Ratio of *Ae*. *aegypti* to *Ae*. *albopictus* mosquitoes caught per week by study arm. Weekly counts of *Ae*. *aegypti* and *Ae*. *albopictus* were aggregated across 12 *w*Mel-treated clusters and 12 untreated clusters. Releases of *w*Mel-infected *Ae*. *aegypti* mosquitoes occurred from March 2017 –December 2017 (yellow shading). The first nine weeks of releases are excluded from analyses due to unequal trapping density in the treated and untreated clusters. There was an average of 4 BG traps/km^2^ in *w*Mel-treated clusters and 5 BG traps/km^2^ in untreated clusters in the pre-release period, and 16 BG traps/km^2^ and 15 BG traps/km^2^ during and post-release.

The *Ae*. aegypti to *Ae*. *albopictus* ratio in each study arm over time is shown in Fig 5 and demonstrates not only the much greater relative prevalence of *Ae*. *aegypti* but also the seasonal “dry season” peaks in this ratio. The ratio of *Ae*. *aegypti* to *Ae*. *albopictus* did not measurably differ between *w*Mel-treated or untreated arms of the study (p = 0.06). The weekly abundance of *Ae*. *aegypti* and *Ae*. *albopictus* in each of the 24 clusters between March 2015 and March 2020 is plotted in S3 and S4 Figs.

### Insecticide resistance profile of mosquito populations in the AWED study area

**Fig 6:**
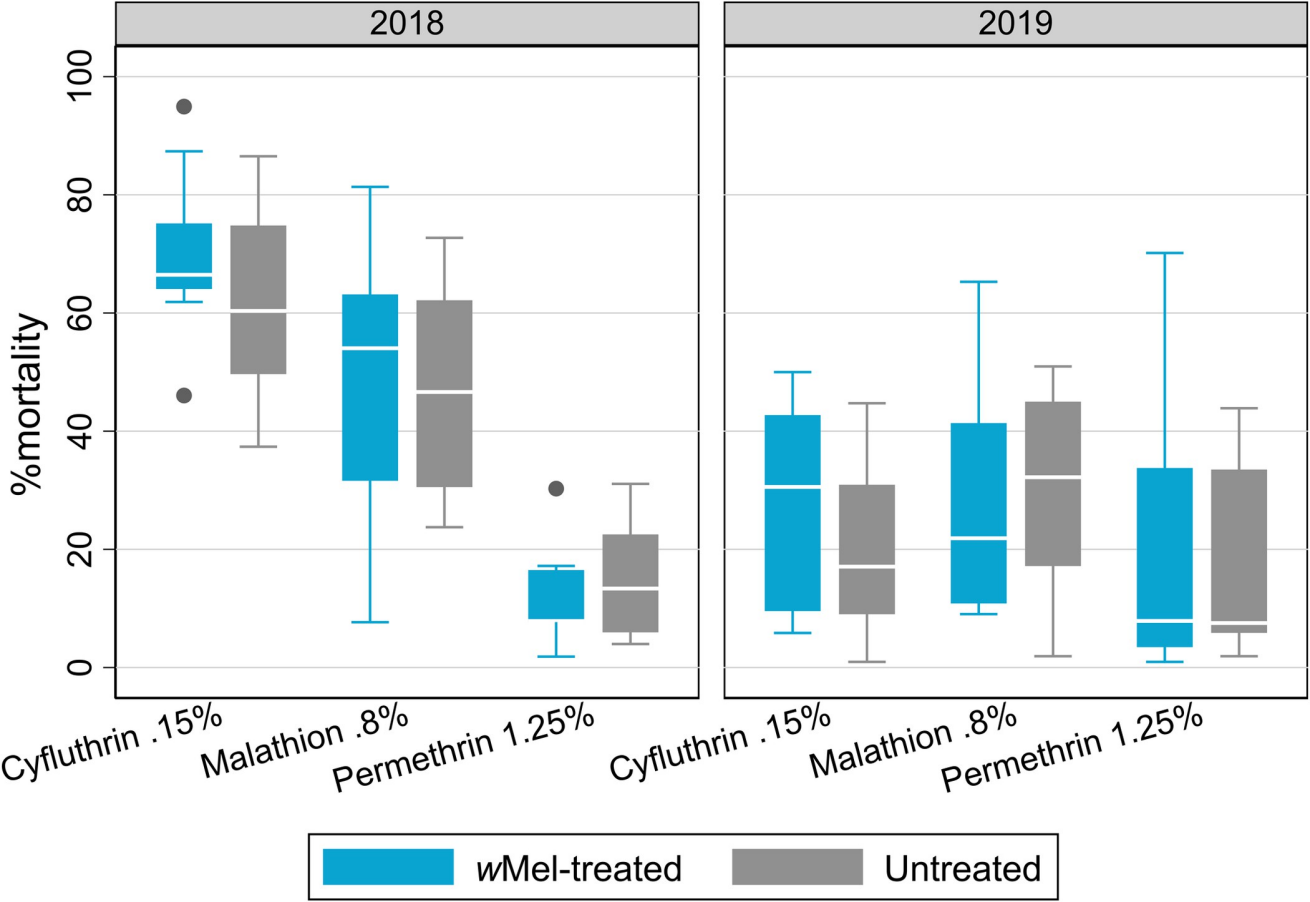
Percentage mortality of *Ae*. *aegypti* in the WHO bioassay for various insecticides by study arm in 2018 and 2019. Boxes are median and interquartile range for percentage mortality in cohorts of mosquitoes derived from 12 *w*Mel-treated clusters and 12 untreated clusters. Whiskers indicate the range, and circles indicate outliers. Insecticide type and concentrations used were in line with recommendations for *Ae*. *aegypti* mosquitoes and followed the WHO standard method [18]. The median (25^th^– 75^th^ percentile) percentage mortality for the susceptible strain (Rockefeller) in 2019 is as follows: 100 (100–100) for cyfluthrin 0.15%; 91 (76–99) for malathion 0.8%; and 100 (97–100) for permethrin 1.25%.

Insecticide resistance testing by bioassay was performed in 2018 and 2019 on field-derived adults sourced from each of the 24 clusters. There was no measurable difference in susceptibility between *Ae*. *aegypti* from the *w*Mel-treated clusters compared to the untreated clusters in either 2018 or 2019, for any of cyfluthrin (0.15%) (p = 0.16 and 0.39, respectively), malathion (0.8%) (p = 1.00 and 0.77) or permethrin (1.25%) (p = 0.71 and 0.73) (Fig 6).

## Discussion

The AWED trial previously reported a 77% (95% CI 65, 85%) reduction in the incidence of virologically-confirmed dengue, and an 86% (95% CI, 66.2 to 94.3) reduction in dengue hospitalisations, in *w*Mel-intervention versus control clusters [8]. The reduced vector competence of *Ae*. *aegypti* in *w*Mel intervention clusters is the simplest explanation for the epidemiological findings in the AWED trial but it was important to consider other possibilities. The results reported here provide evidence that the trial’s epidemiological findings were likely not confounded by differences in *Ae*. *aegypti* adult abundance or insecticide resistance characteristics between the two study arms. Additionally, the relatively low abundance of *Ae*. *albopictus*, and the balance in numbers between study arms, suggests the trial outcomes were also not confounded by this secondary vector.

A body of literature, albeit mostly from laboratory studies, has described *w*Mel-infection as having negligible or small fitness costs in one or more mosquito life history traits. For example, in laboratory conditions Walker *et al* described no impact of *w*Mel on adult fecundity, egg survivorship or larval development time, but did report reduced (~10%) adult female survivorship times compared to controls [5]. Conversely, Fraser *et al* [12] described no impact of *w*Mel on adult longevity but did find egg survival times were slightly reduced compared to wild-type counterparts. Ross *et al* reported that *w*Mel-infection reduced larval survival by ~15% under strict starvation conditions that pressure tested the nutritional reserves of instar first and second larval instars [19]. Using field-derived adult mosquitoes, Hoffmann *et al* measured ~25% lower fecundity and larvae production from *w*Mel-infected females when compared to wild-type counterparts [20]. In Brazil, Farnesi *et al* [11] found that *w*Mel infection delayed embryogenesis, decreased desiccation resistance through delayed eggshell formation and eggs had substantially lower survivorship after 6 weeks storage compared to uninfected control mosquitoes. Why then did we find no differences in the abundance of *Ae*. *aegypti* adults in intervention clusters versus control clusters? One possible explanation is that the collective fitness costs of *w*Mel in the field are too small to manifest as a measurable difference in *Ae*. *aegypti* counts between treatment arms, despite 60,084 BG trap collections and 478,254 *Ae*. *aegypti* caught. Our power estimates suggested 80% power to detect a 20% difference or greater in the number of *Ae*. *aegypti* between *w*Mel intervention and control clusters, suggesting that if there is a true difference in relative abundance it is probably smaller than 20%.

Globally, *Ae*. *albopictus* is considered a secondary vector of dengue relative to *Ae*. *aegypti*. In Yogyakarta, *Ae*. *aegypti* was far more prevalent than *Ae*. *albopictus* in the BG trap network in all 24 trial clusters and there was no difference in *Ae*. *albopictus* abundance between study arms. This is encouraging from the perspective that *w*Mel introgression into *Ae*. *aegypti* did not lead to any measurable alteration in the ecological balance between the two species that might inadvertently allow *Ae*. *albopictus* to expand its population size in Yogyakarta. The much lower abundance of *Ae*. *albopictus* relative to *Ae*. *aegypti* is partly explained by Yogyakarta being mostly a highly urbanised environment that favours *Ae*. *aegypti’s* domesticated lifestyle. The role of *Ae*. *albopictus* in the epidemiology of dengue in Yogyakarta is unclear. Going forward, it will be important to explore the relative contributions of *Ae*. *albopictus* versus *w*Mel-infected *Ae*. *aegypti* in any ongoing dengue transmission that occurs in Yogyakarta after city-wide *w*Mel introgression is completed in 2021.

In Indonesia, organophosphates (temephos and malathion) and pyrethroids are the two major classes of insecticide utilized for *Aedes* control. Cyfluthrin (a pyrethroid) and malathion are used by Yogyakarta authorities in fogging activities in response to dengue cases in the community. Resistance to pyrethroids was documented in *Ae*. *aegypti* from Yogyakarta City in 2015 [21]. To help ensure the competitiveness of the *w*Mel release material deployed in the AWED trial the insecticide resistance profile of the mosquito line was matched to the wild-type field population by repeated outcrossing with wild-type mosquitoes from Yogyakarta City [8]. It was reassuring therefore that we found equivalent insecticide resistance profiles between cohorts of mosquitoes from the *w*Mel treated clusters versus the untreated clusters in 2018 and again in 2019. This provides evidence that the release material used in 2017 was suitably matched to the local insecticide resistance profile. It also provides further reassurance that the epidemiological effect measured in the AWED trial was not confounded by a difference in the sensitivity of mosquitoes to insecticides between the two study arms. We note however that WHO insecticide resistance bioassays are semi-quantitative in nature and that small differences in absolute insecticide resistance are difficult to measure. It will be of great interest to track the insecticide resistance profile of the *Ae*. *aegypti* in Yogyakarta over coming years. One hypothesis is that dengue case incidence will be very low in Yogyakarta after city-wide establishment of *w*Mel and that this will significantly decrease insecticide fogging episodes carried out by local authorities. In turn, this diminished selective pressure might lead to a degree of restoration of insecticide susceptibility in the mosquito population.

Our study had some limitations. We did not measure variables such as household-level insecticide usage or local community behaviours that might have been confounding to the trial results, though we think that randomisation will have balanced these parameters. Our methods of mosquito surveillance (BG traps located predominantly indoors) were plausibly biased to detecting *Ae*. *aegypti* rather than *Ae*. *albopictus*. Indeed, *Ae*. *albopictus* should be taken into consideration when interpreting dengue epidemiology in Yogyakarta from 2021 onwards.

Collectively, the results here suggest *w*Mel introgression to a high and stable prevalence had no measurable impact on adult *Ae*. *aegypti* population size or insecticide resistance phenotype. The results will be reassuring to audiences concerned about ecological impacts of *w*Mel introgression. More generally, we’d expect the *w*Mel introgression method to deliver similar positive public health benefits and the same unremarkable entomological outcomes in other dengue-endemic urban settings in Indonesia.

## Supporting information

S1 Table BG trap distribution per cluster. (DOCX)

S1 Fig BG trap network used to monitor *w*Mel establishment in the post-intervention period. Each point on the map shows the location of a BG trap in the post-intervention time period of January 2018—March 2020. This map was created using ArcGIS software by ESRI. The vector map was sourced from the local government (Regional body for planning and development) and ground-truthed by the study team. The shapefile for the map can be found at https://doi.org/10.6084/m9.figshare.19450415.v1.
(TIF)

S2 Fig Monthly *w*Mel positivity in *Ae*. *aegypti* mosquitoes caught in- A) *w*Mel-treated clusters and B) untreated clusters. In panel A, the yellow shading represents the *w*Mel-infected mosquito release period for each cluster.
(TIF)

S3 Fig *Ae*. *aegypti* mosquitoes caught per trap per week in A) *w*Mel-treated clusters and B) untreated clusters. Releases of *w*Mel-infected *Ae*. *aegypti* mosquitoes occurred from March 2017 –December 2017 (yellow shading). The first nine weeks of releases are excluded from analyses due to unequal trapping density in the treated and untreated clusters. There was an average of 4 BG traps/km^2^ in *w*Mel-treated clusters and 5 BG traps/km^2^ in untreated clusters in the pre-release period, and 16 BG traps/km^2^ and 15 BG traps/km^2^ during and post-release.
(TIF)

S4 Fig *Ae*. *albopictus* mosquitoes caught per trap per week in A) *w*Mel-treated clusters and B) untreated clusters. Releases of *w*Mel-infected *Ae*. *aegypti* mosquitoes occurred from March 2017 –December 2017 (yellow shading). The first nine weeks of releases are excluded from analyses due to unequal trapping density in the treated and untreated clusters. There was an average of 4 BG traps/km^2^ in *w*Mel-treated clusters and 5 BG traps/km^2^ in untreated clusters in the pre-release period, and 16 BG traps/km^2^ and 15 BG traps/km^2^ during and post-release.
(TIF)

S1 Data Mean weekly counts of *Ae*. *aegypti* and *Ae*. *albopictus* mosquitoes caught per trap by study arm and percentage mortality of *Ae*. *aegypti* for various insecticides by study arm in 2018 and 2019. (XLSX)
