# Supplementary material for: Aedes aegypti abundance and insecticide resistance profiles in the Applying Wolbachia to Eliminate Dengue trial
Source: PLoS Negl Trop Dis. 2022 Apr 20;16(4):e0010284. doi: 10.1371/journal.pntd.0010284 (PMC9060332; doi:10.1371/journal.pntd.0010284)
Supplement: S1 Table — (DOCX) [file pntd.0010284.s001.docx]

**S1 Table.** **BG trap distribution per cluster**

|  | Cluster area (total, km^2^) | Cluster area (release, km^2^) | No. BG traps* | BG trap density* | Nearest Neighbour Index† | Mean distance to nearest trap† (metres) | Minimum distance of any trap from cluster boundary† (metres) |
| --- | --- | --- | --- | --- | --- | --- | --- |
| *Wolbachia Intervention* | | | | | | | |
| Cluster 1 | 0.94 | 0.88 | 14 | 15.9 | 1.70 | 217.41 | 39.82 |
| Cluster 2 | 0.69 | 0.69 | 10 | 14.5 | 1.39 | 185.01 | 61.26 |
| Cluster 6 | 0.94 | 0.93 | 14 | 15.1 | 1.61 | 211.22 | 31.55 |
| Cluster 7 | 1.11 | 0.98 | 16 | 16.3 | 1.51 | 199.28 | 69.01 |
| Cluster 9 | 1.14 | 1.06 | 14 | 13.2 | 1.51 | 210.78 | 78.93 |
| Cluster 10 | 1.17 | 1.00 | 16 | 16.0 | 1.87 | 237.57 | 69.55 |
| Cluster 12 | 1.13 | 1.08 | 18 | 16.7 | 1.68 | 211.43 | 52.03 |
| Cluster 14 | 1.27 | 1.14 | 17 | 14.9 | 1.58 | 211.74 | 69.76 |
| Cluster 16 | 0.89 | 0.86 | 14 | 16.3 | 1.59 | 199.67 | 83.56 |
| Cluster 19 | 0.83 | 0.83 | 13 | 15.7 | 1.84 | 234.44 | 74.81 |
| Cluster 21 | 1.04 | 0.87 | 14 | 16.1 | 1.47 | 186.73 | 51.56 |
| Cluster 24 | 1.09 | 0.72 | 13 | 18.1 | 1.89 | 238.94 | 66.49 |
| *Control* | | | | | | | |
| Cluster 3 | 1.02 | 0.92 | 15 | 16.3 | 1.74 | 219.41 | 72.46 |
| Cluster 4 | 0.93 | 0.73 | 11 | 15.1 | 1.75 | 231.60 | 51.59 |
| Cluster 5 | 1.10 | 1.06 | 13 | 12.3 | 1.64 | 237.67 | 41.64 |
| Cluster 8 | 1.12 | 1.08 | 16 | 14.8 | 1.77 | 229.84 | 45.81 |
| Cluster 11 | 0.86 | 0.80 | 10 | 12.5 | 1.74 | 241.96 | 68.83 |
| Cluster 13 | 1.64 | 1.54 | 24 | 15.6 | 1.69 | 217.45 | 62.86 |
| Cluster 15 | 0.95 | 0.87 | 9 | 10.3 | 1.81 | 280.38 | 67.06 |
| Cluster 17 | 0.73 | 0.63 | 10 | 15.9 | 1.57 | 203.46 | 79.17 |
| Cluster 18 | 1.60 | 1.43 | 24 | 16.8 | 1.65 | 207.97 | 73.05 |
| Cluster 20 | 1.03 | 1.01 | 16 | 15.8 | 1.65 | 209.88 | 68.1 |
| Cluster 22 | 1.33 | 1.16 | 15 | 12.9 | 1.52 | 211.72 | 73.11 |
| Cluster 23 | 1.07 | 0.90 | 12 | 13.3 | 1.90 | 249.26 | 101.74 |

*as per BG trap network in place between January 2018 - March 2020

†as per BG trap network in place between January 2019 - March 2020. Number of BG traps per cluster remained unchanged throughout the post-intervention period (January 2018 - March 2020), however, some traps moved locations within clusters when agreements with host households expired and were unable to be renewed.
