# Supplementary figures and images for: Aedes aegypti abundance and insecticide resistance profiles in the Applying Wolbachia to Eliminate Dengue trial
Source: PLoS Negl Trop Dis. 2022 Apr 20;16(4):e0010284. doi: 10.1371/journal.pntd.0010284 (PMC9060332; doi:10.1371/journal.pntd.0010284)

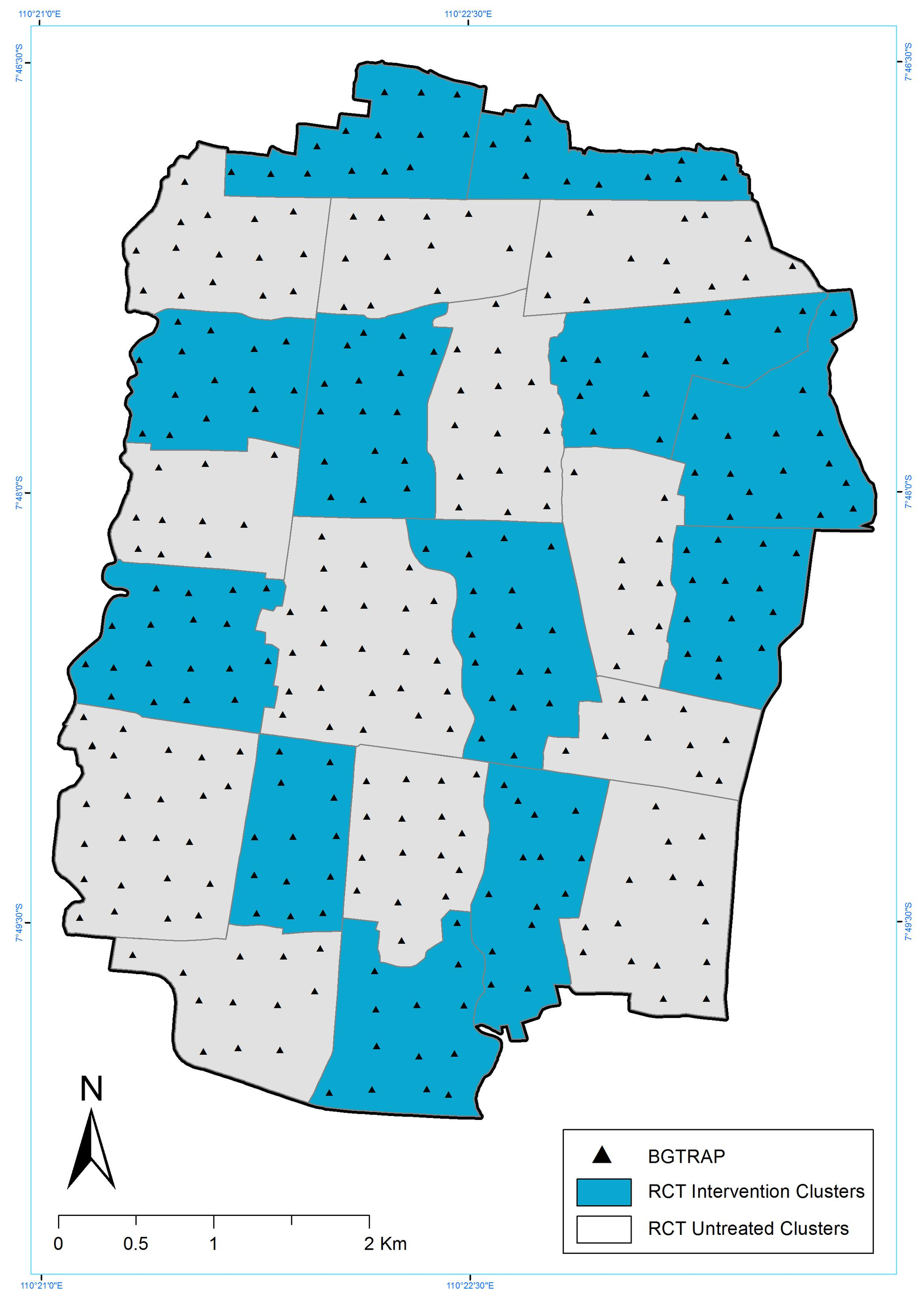

Supplement: S1 Fig — Each point on the map shows the location of a BG trap in the post-intervention time period of January 2018—March 2020. This map was created using ArcGIS software by ESRI. The vector map was sourced from the local government (Regional body for planning and development) and ground-truthed by the study team. The shapefile for the map can be found at https://doi.org/10.6084/m9.figshare.19450415.v1. (TIF) [file pntd.0010284.s002.tif]

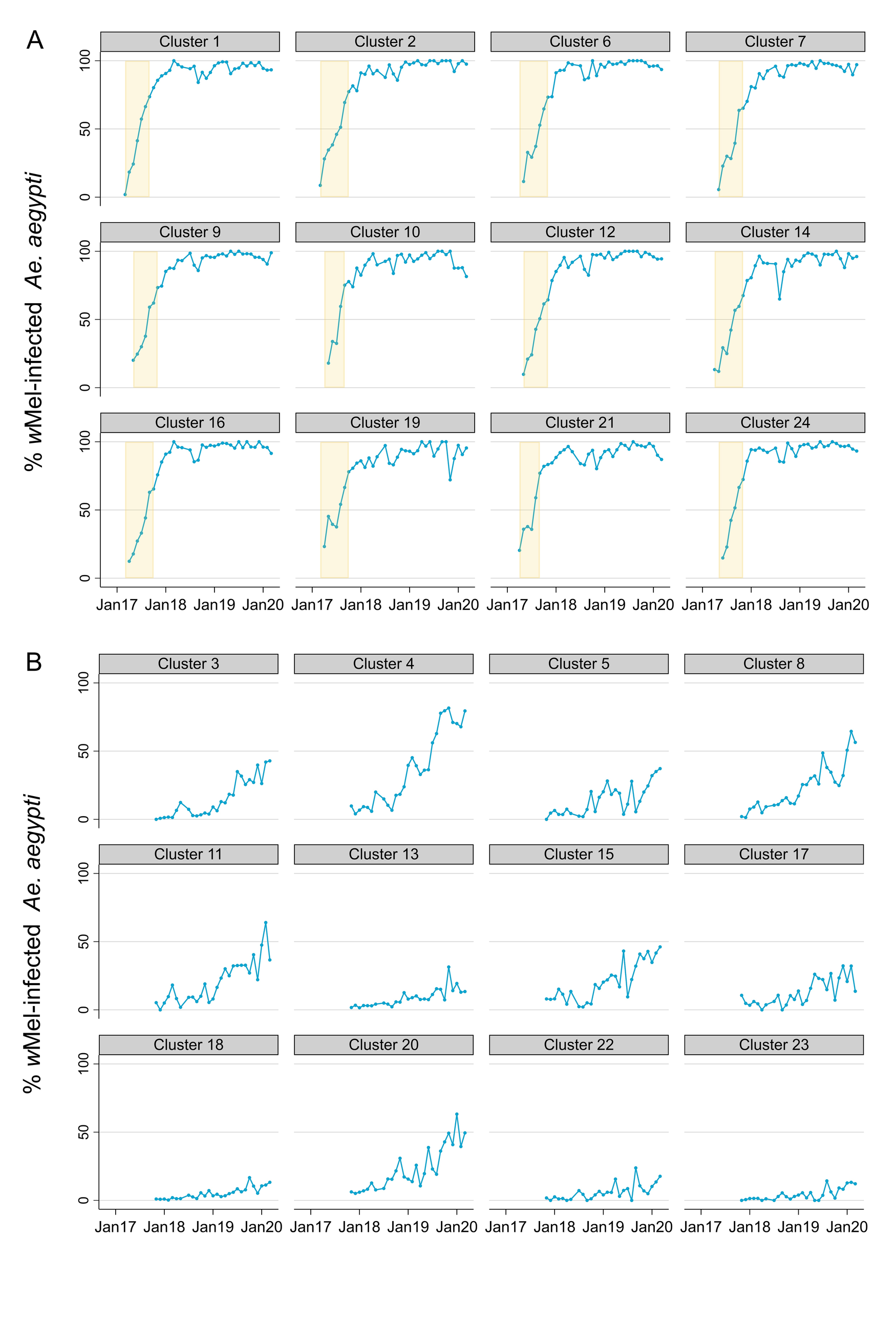

Supplement: S2 Fig — In panel A, the yellow shading represents the wMel-infected mosquito release period for each cluster. (TIF) [file pntd.0010284.s003.tif]

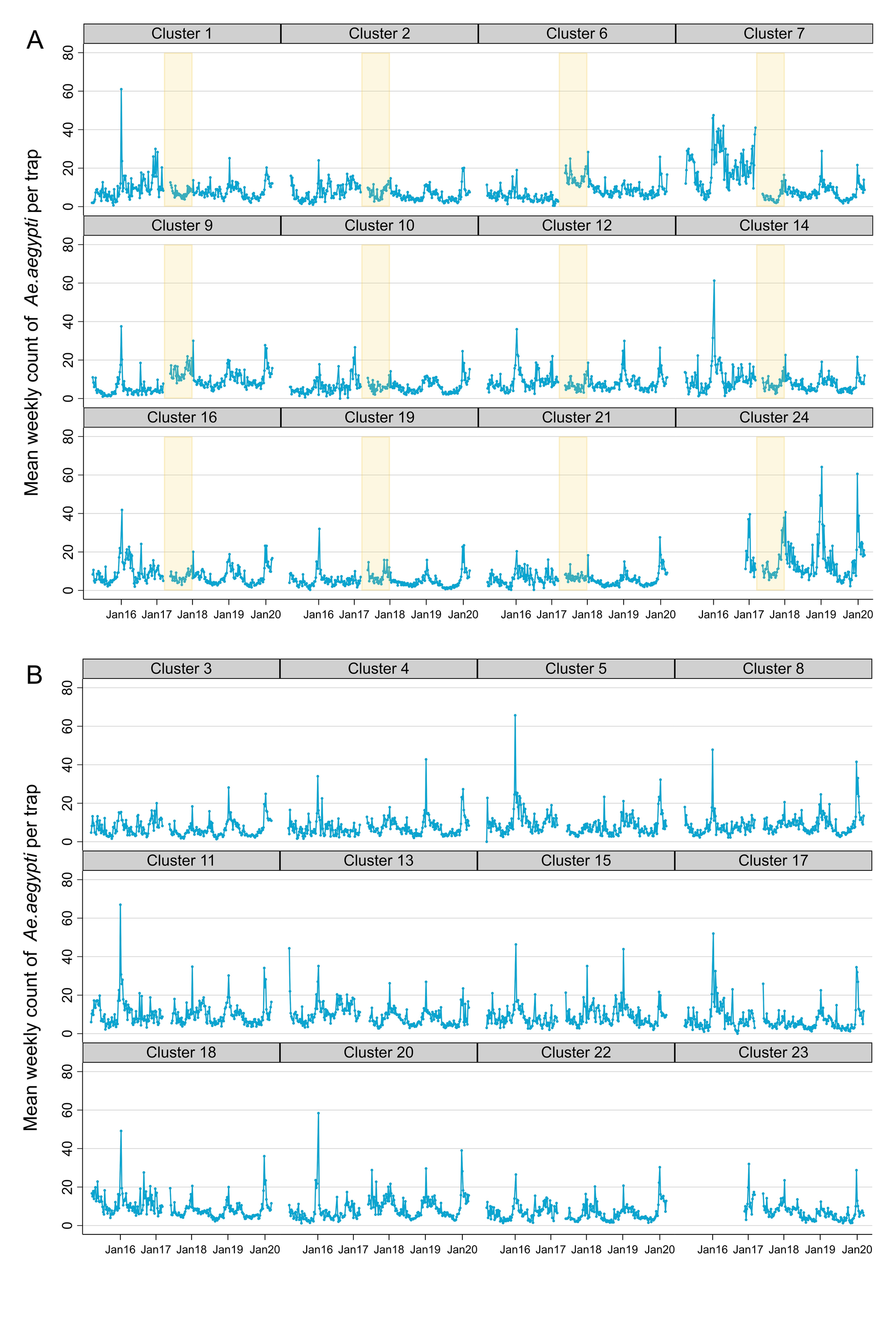

Supplement: S3 Fig — Releases of wMel-infected Ae. aegypti mosquitoes occurred from March 2017 –December 2017 (yellow shading). The first nine weeks of releases are excluded from analyses due to unequal trapping density in the treated and untreated clusters. There was an average of 4 BG traps/km2 in wMel-treated clusters and 5 BG traps/km2 in untreated clusters in the pre-release period, and 16 BG traps/km2 and 15 BG traps/km2 during and post-release. (TIF) [file pntd.0010284.s004.tif]

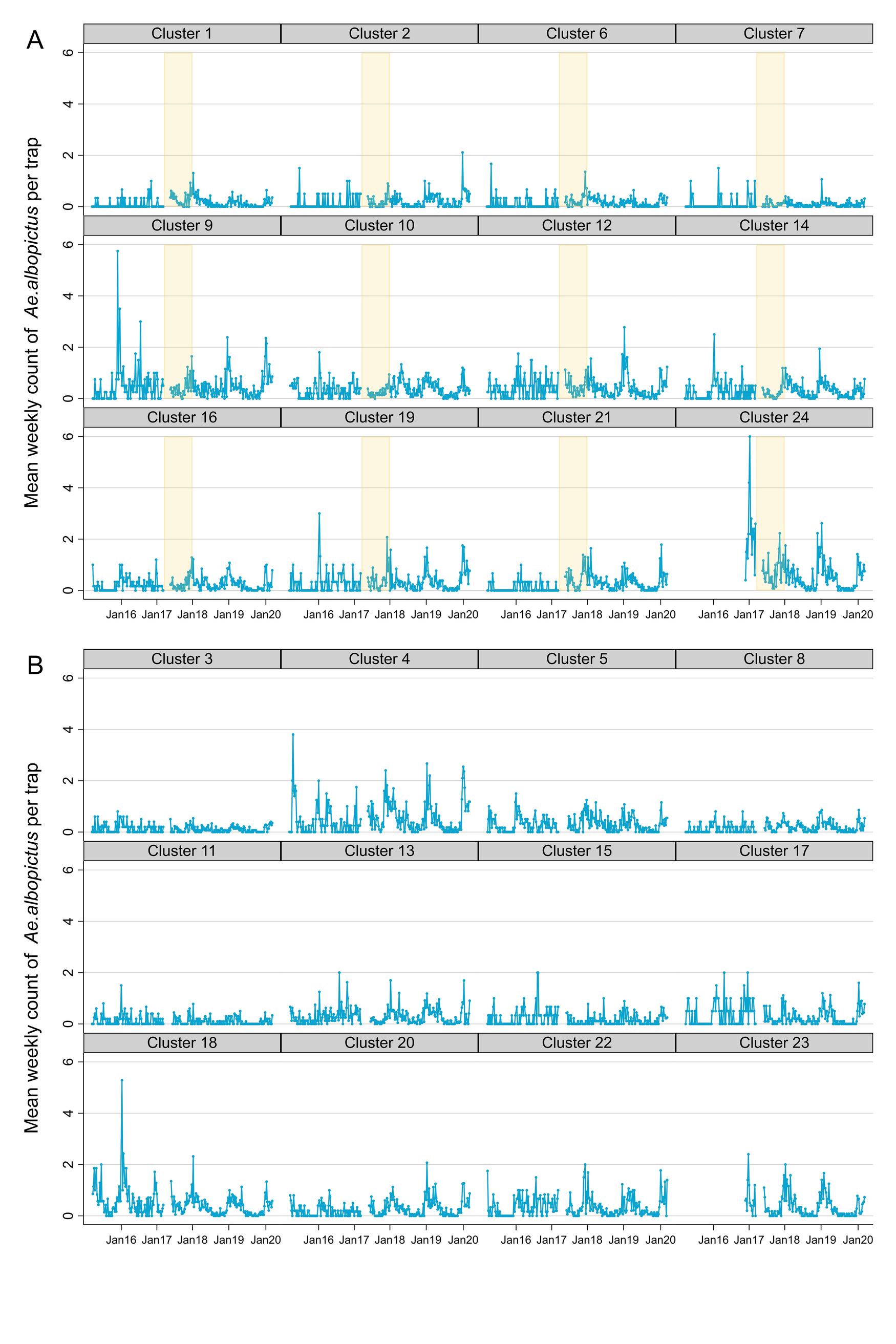

Supplement: S4 Fig — Releases of wMel-infected Ae. aegypti mosquitoes occurred from March 2017 –December 2017 (yellow shading). The first nine weeks of releases are excluded from analyses due to unequal trapping density in the treated and untreated clusters. There was an average of 4 BG traps/km2 in wMel-treated clusters and 5 BG traps/km2 in untreated clusters in the pre-release period, and 16 BG traps/km2 and 15 BG traps/km2 during and post-release. (TIF) [file pntd.0010284.s005.tif]
